# Safety & Efficacy of Cyclic Zoledronic Acid Therapy on Pediatric Secondary Osteoporosis

**DOI:** 10.5539/gjhs.v8n8p20

**Published:** 2015-12-17

**Authors:** Abdulmoein E. Al-Agha, Talal A. Shaikhain, Abdullah A. Ashour

**Affiliations:** 1 Faculty of Medicine, King Abdulaziz University, Jeddah, Kingdom of Saudi Arabia; 2 Department of Pediatrics, Faculty of Medicine, King Abdulaziz University, Jeddah, Kingdom of Saudi Arabia

**Keywords:** multiple morbidities, secondary osteoporosis, Zoledronic acid

## Abstract

**Background/Aim:**

Osteoporosis is a systemic disease characterized by decreased bone density and increased tendency to develop fractures. Osteoporosis in children and adolescents is a rare disease usually secondary to Medical conditions or medications given to children. The condition affects normal bone growth and development and carries with it multiple morbidities (physical and psychological) if not corrected promptly. This study aims to share our experience with Zoledronic Acid Therapy in Pediatric patients with secondary osteoporosis.

**Method:**

A retrospective study which included 46 patients aged 3 to 18 years. All patients received specific doses of Zoledronic acid and were followed up at King Abdulaziz University Hospital (KAUH) in Jeddah, Saudi Arabia. Clinical and laboratory data were collected for each patient from their files. Adverse events were also recorded.

**Results:**

The use of Zoledronic Acid in children and adolescents appears to be statically significant reduce fracture rate (p=0.005), bone turnover markers (Osteocalcin p= 0.003, CTX p= 0.008) and pain frequency in symptomatic individuals (p=0.000). Careful selection of cases is required to provide maximum benefits compared to risks associated with therapy.

**Conclusion:**

This study demonstrates that Zoledronic acid has positive effects on clinical outcome and bone marker level as well as quality of life for Pediatric patients with Osteoporosis and their families, with no long-term side effects.

## 1. Introduction

Osteoporosis is a systemic skeletal disease characterized by decreased in bone mineral density which lead to decrease bone strength and increase risk of fracture (Bachrach & Ward, 2009; Ward, 2010). Pediatric osteoporosis can be diagnosed clinically by expert physician in patient with a significant fracture (even if the other investigation is normal) and age-adjusted Z-scores ≤ -2.0 SD in Dual-Energy X-ray absorptiometry (DXA.) (Bishop et al., 2014; Ooi, Briody, McQuade, & Munns, 2012; Ward, 2010).

Childhood and adolescence are in a critical period for skeletal maturity and bone strength and osteoporosis in this period might have life-long effects. Bone strength is determined by bone size, quality, geometry and density and these variables are influenced by genetic factors, hormones, nutrition and physical activity (Karlsson, Nordqvist, & Karlsson, 2008; Ooi et al., 2012). Lifetime risk of osteoporosis increases by compromised bone strength in children (“children” used in wider range including young individual up to 18 years old) with genetic skeletal disorder or chronic disease (Bianchi, 2007; Papapoulos, 2011). The severity of chronic diseases and age of onset are associated with increased incidence of the osteoporosis (Bianchi, 2007). Children with chronic diseases have increased risk of fragile fracture either secondary to underlying conditions that adversely affect skeletal development (e.g. reduce mobility, malabsorption, poor nutrition, metabolic or hormonal abnormality, vitamin D deficiency or inflammation) and/or because of medications adverse effects (e.g. glucocorticoids) (Bachrach & Ward, 2009; Bianchi, 2007; Makitie, 2013).

Osteoporosis treatment aiming to increase bone density and bone quality in order to reduce fracture risk and subsequently improve the quality of life (Makitie, 2013). Bisphosphonates are synthetic component similar to pyrophosphate which has anti resorption effect by interfering with osteoclast attachment to the bone surface and induce osteoclast apoptosis, and also promote bone formation by stimulating osteoblastic activity (Carpenter et al., 2007; Makitie, 2013; Nanni, 2005). Over 30 years bisphosphonates were the major drugs used in adult to treat hyperresorption bone disease although, in pediatric it’s not yet approved by FDA for lack of data about safety and efficacy of bisphosphonates (Makitie, 2013; Ooi et al., 2012). In adult, bisphosphonates are shown to be safe and are first-choice agents for more than 10 years in osteoporosis management, especially in postmenopausal and secondary causes of osteoporosis (Bachrach & Ward, 2009; Reid, 2011). Other aspects of management of secondary osteoporosis include: increase physical activity, adequate mobilization and weight bearing activities, ensuring adequate vitamin D and calcium intake, improve general nutrition, minimizing osteotoxic medication as possible and decreasing risk of accidental trauma (Munns & Cowell, 2005). In this study, we aimed to review our experience of using Zoledronic acid therapy in children and adolescents with secondary osteoporosis (in Jeddah, Saudi Arabia).

## 2. Methodology

### 2.1 Patients

Forty-nine patients who took Zoledronic acid between the years 2002 and 2014 were interviewed. The sample consisted of children and adolescents with secondary osteoporosis. They were selected by expert pediatric endocrinologist on the basis of their clinical presentations (significant low trauma bone fractures, bone pain and physical disability) with high bone turnover markers. All patients were having normal nutrition, calcium, phosphate and vitamin D status. Moreover, the study had its own pediatric endocrine outpatient clinic in KAUH, to confirm the diagnosis. The molecular screening for checking the Bone Mineral Density (BMD) measurement for young children was not available at the clinical institute.

This study assessed the clinical efficacy of Zoledronic acid in patients who had completed at least 6 months of Zoledronic drug treatment any changes in their quality of life, mobility and bone pain. The selected study group consisted of osteoporotic children and adolescents aged between 3-18 years when treatment started. All of the selected participants appeared to have normal bone profile, vitamin D and Parathyroid Hormone (PTH) before the initiation of the treatment. Zoledronic acid, a bisphosphonate has been used as the treatment agent in this research based study.

### 2.2 Treatment

The legal and ethical code of conduct was maintained throughout the time period of the study. A written consent was taken from all the patients and/or their parents prior to the commencement of the Zoledronic acid therapy. Zoledronic acid powder required reconstitution with 5 ml sterile water, before its infusion to the patients. The reconstituted solution was then diluted in 50 ml normal saline. A dose of 0.05 mg/kg of Zoledronic acid (Zometa^®^) were given intravenously to the patients over the span of 60 minutes. The maximum dose to be given is 2mg/kg infusion over 60 minutes. In case of neonates and those less than one year, the dose was decreased down to 0.025 mg/kg. The first five infusions were given once every three months. Afterward, the doses were given once every six months, depending on clinical and biochemical markers response.

All the patients were admitted for two days for the first infusion, in order to closely monitor the acute complications that might occur. The complications that were checked in patients were fever, myalgia, hypocalcemia, flu-like symptoms, and bone pain. Subsequent Zoledronic acid infusions were given during day care admissions. To avoid hypocalcemia, all the patients were given a continuous calcium infusion of 200-400 mg/kg per day together with their first dose of Zoledronic infusion. All patients were given adequate oral calcium intake, a daily dose of 1200 mg. Moreover, a daily dose of prophylactic Vitamin D (400-800 IU) was routinely provided as well. Ibuprofen, 10 mg/kg, was also administered three to four times per day during the first 2 days following the first infusion. Ibuprofen was given to minimize fever and myalgia that was frequently observed in patients.

### 2.3 Assessment

#### 2.3.1 Anthropometric Measurements

Body height and weight were measured for all patients in the study. Age and sex matched standardized scores (SDs) were measured by the help of Children’s Hospital Boston Growth Calculator 2.01 (Hamill et al., 1979).

#### 2.3.2 Clinical Assessment

Clinical assessment was carried out by medical doctors on patients and/or their parents during the follow-up appointment in the clinic in a form of questionnaire. The questionnaire consisted of questions which compared the symptoms before and after starting intravenous Zoledronic acid treatment. These symptoms included bone pain and its site (generalized, lower back, hip, neck, and/or upper or lower extremities) and estimated frequency of pain (daily, weekly defined as pain more than one day per week, or infrequently defined as less than one day per week). Mobility (ranging from immobile, moving from side to side, crawling, walking with limitation, limping or using walking aid, or walking) was assessed during the 2 years of treatment, to rule out any confounding factor from normal development milestone, and exercise type and its frequency.

Fracture rate per year was calculated by dividing number of fractures prior the initiation of treatment over number of years from the first fracture (Hamill et al., 1979). For those in which treatment started before 1 year of age, fracture rate was calculated by dividing the number of fracture by the duration in months and then multiplying it by 12. The quality of life was also assessed subjectively by asking the patient or parents if it was normal or below normal compared to their peers of same age, and whether it improved after the treatment, stayed the same or got worse.

#### 2.3.3 Assessment of Side Effects

Acute side effects that were reported after the first dose of Zoledronic acid infusion included fever, hypocalcemia, decreased oral intake, bone pain, myalgia, and flu-like symptoms. Moreover, chronic nephrocalcinosis and renal failure were assessed by renal ultrasound done as baseline before starting the treatment and 1 year after. The renal profile at baseline, before and after each infusion cycle was checked for any kind of complications. Patients with creatinine clearance lower than 30-35 ml/min are usually prohibited from bisphosphonate treatment. Further, if any patient had mineral metabolism disturbance, then it must be corrected before the administration of the bisphosphonates in any bone-related disorder. In addition to this, patients who have been under treatment for five years are recommended to not prolong the treatment for more than five years since bisphosphonates reside in bones. If no fractures occurred after the provision of treatment, then it is advised that the treatment should no longer be continued in order to avoid any kind of implications from the drug intake (Watts & Diab, 2010).

#### 2.3.4 Laboratory Assessment

Serum levels of calcium, 25-hydroxyl vitamin D, phosphate, parathyroid hormone (PTH) and alkaline phosphatase were measured. Moreover, creatinine levels were also assessed as a baseline before the infusion of each Zoledronic acid dose. In this study, a normal value of serum calcium of 2.12-2.52 mmol/L was adopted. The study categorized the severity of hypocalcemia according to the following calcium levels; mild 1.8-2.11 mmol/L, moderate 1.5-1.7 mmol/L, severe < 1.5 mmol/L. Normal levels of 25-hydroxyl (OH) vitamin D was considered to be 50-80 nmol/L. Bone markers including serum osteocalcin and CTX were measured at baseline before starting the treatment and again after starting treatment every 3 to 6 month according to treatment period. Serum osteocalcin normal values in females ranged between 11-43 ng/ml while in males it ranged between 24-70 ng/ml. Normal serum CTX values in females were considered less than 0.57 ng/ml while in males less than 0.58 ng/ml.

#### 2.3.5 Radiological Assessment

There was a limitation in Bone Mass Density (BMD) assessments as hospital’s DXA scan machine software didn’t support measuring bone density for patients below the age of 5 years as well the frequent machine dysfunction. Also, some baseline DXA went missing as it was done outside the hospital for many of the patients. It was decided not to include any radiological data in this study and we provide some of them in (Table 5).

**Table 1:** Detailed clinical characteristics of the population

Number	Age	Age of Dx	Age of Tx	Gender	Height	Height SD	No. fracture bef.	No. fracture aft.	Number of doses
1	15	14	14	Male	168	-0.36	N/A	N/A	20
2	13	N/A	N/A	Female	138	-2.93	N/A	N/A	1
3	23.17	N/A	N/A	Male	170	OAR	N/A	N/A	10
4	11	N/A	N/A	Female	120	-3.51	N/A	N/A	5
5	14	N/A	14	Male	140	0.7	N/A	N/A	3
6	20	N/A	10	Female	150	OAR	N/A	N/A	26
7	14.42	N/A	14	Male	120	-6.14	N/A	N/A	15
8	21	12	11.9	Female	N/A	N/A	N/A	N/A	17
9	16.33	N/A	N/A	Female	151.5	-3.24	3	0	29
10	15.67	3	13	Female	122	-6.16	2	0	6
11	18.58	6	14	Male	152	OAR	2	0.2	10
12	18	17	18	Female	140	OAR	2	0	3
13	9	8	8	Male	128	-1.03	1	0	5
14	13	11	12	Female	125	-4.85	1	0	2
15	14	13	13	Female	95	-9.93	1	0	1
16	4.83	2	8	Male	91.5	-3.64	1	0	4
17	12.42	3	3	Male	120	-4.17	0.67	0.11	29
18	11	3	11	Male	133	-1.79	0	0	14
19	12.9	10	12.9	Female	157	0.01	0	0	1
20	16.58	16	N/A	Female	157	-0.91	0	0	5
21	13.67	13	N/A	Male	156	-0.9	0	0	1
22	12.75	12	N/A	Female	141	-2.05	0	0	2
23	17.17	7	7	Male	168	-1.32	0	0	23
24	5	N/A	N/A	Male	112	0.36	0	0	1
25	8.58	8.17	8.25	Male	135	0.87	0	0	2
26	12.92	N/A	N/A	Female	141.5	-2.17	0	0	1
27	19.08	17	17	Male	146.1	OAR	0	0	3
28	15.42	15	N/A	Male	147	-2.51	0	0	1
29	15.17	12	12	Female	152.5	-1.49	0	0	20
30	7.92	2	6	Female	98	-4.77	0	0	5
31	10.58	10	N/A	Female	116	1.27	0	0	1
32	21.42	13	14	Female	199	OAR	0	0	15
33	16.83	16.83	16.83	Female	158	-0.79	0	0	3
34	13.08	N/A	N/A	Female	145.1	-1.76	0	0	1
35	19.83	8	10	Female	119.5	OAR	0	0	21
36	19.83	8	10	Female	126	OAR	0	0	25
37	9.33	9	N/A	Female	117.5	-2.52	0	0	3
38	18.67	16	16	Male	141	OAR	0	0	6
39	16.5	14.25	N/A	Female	150	-2.01	0	0	16
40	24.83	15	17	Male	159	OAR	0	0	10
41	24	14	N/A	Female	145.2	OAR	0	0	1
42	12.33	12	N/A	Female	136	-2.43	0	0	2
43	17	3	3	Female	130	-5.16	0	0	17
44	11	5	5	Male	131.5	-1.7	0	0	16
45	10	9	N/A	Female	133	-0.7	0	0	2
46	12	11	N/A	Female	138	-2.09	0	0	1

*Note*. Dx= Diagnosis, SD=Standard Deviation.

**Table 2:** Causes of secondary osteoporosis in the population

Percentage	Frequency		
9.2%	12	Hematological Diseases	**Secondary Osteoporosis**
9.2%	12	Inflammatory Bowel Disease	
6.1%	8	Renal Diseases	
6.1%	8	Endocrine Diseases	
3.1%	4	Drug-Induced	
3.1%	4	Immobilization	

**Table 3:** Detailed diagnoses of patients

Percentage	Frequency		
6.1%	8	Sickle cell anemia	Hematological Diseases
3.1%	4	Thalassemia	
4.6%	6	Crohn’s disease	Inflammatory Bowel Disease
1.5%	2	Ulcerative colitis	
3.1%	4	Celiac disease	
3.1%	4	Hypophosphatemic Rickets	Renal Diseases
1.5%	2	Renal failure	
1.5%	2	Renal tubular acidosis	
5.3%	7	Insulin dependent diabetes	Endocrine Disease
0.8%	1	Growth hormone deficiency	
1.5%	2	Steroid + Chemo	Drug-Induced
0.8%	1	Methotrexate	
0.8%	1	GnRH agonist	
2.3%	3	Mucopolysaccharidosis	Immobilization
0.8%	1	Leigh’s disease	

**Table 4:** Pain site & frequency before and after treatment

	Pain Frequency No Pain	Infrequent	Weekly	Daily
Before	10 (26.74%)	12 (42.86%)	5 (17.86%)	11 (39.29%)
After	8 (42.11%)	7 (36.84%)	1 (5.26%)	3 (15.79%)

**Table 5:** 

Site of Pain Generalized	Lower back	Hip	Neck	Lower limb
11 (28.95%)	10 (26.32%)	7 (18.42%)	4 (10.53%)	8 (21.05%)

**Table 6:** Detailed biochemical characteristics of the population

CTX		Osteo	
Pre Rx	Post Rx	Pre Rx	Post Rx
N/A	N/A	N/A	N/A
N/A	N/A	N/A	N/A
N/A	N/A	N/A	N/A
0.59	N/A	28.72	N/A
N/A	N/A	N/A	N/A
N/A	N/A	N/A	N/A
0.27	0.18	53	20.96
0.89	0.35	70.03	44.46
N/A	N/A	N/A	N/A
0.87	0.5	51.4	51.19
N/A	N/A	N/A	N/A
2.7	0.4	214.4	99.7
N/A	N/A	N/A	N/A
N/A	N/A	N/A	N/A
N/A	N/A	N/A	N/A
N/A	N/A	N/A	N/A
N/A	N/A	N/A	N/A
0.58	0.58	75.5	53.5
N/A	N/A	N/A	N/A
N/A	N/A	N/A	N/A
N/A	N/A	N/A	N/A
1.72	N/A	60.6	N/A
1.82	N/A	89.31	N/A
0.58	0.5	39.82	32.07
N/A	N/A	N/A	N/A
1.6	0.8	57.1	41.9
1.52	N/A	130.4	N/A
N/A	N/A	N/A	N/A
N/A	N/A	N/A	N/A
2.12	N/A	120.6	N/A
0.3	N/A	26.6	N/A
N/A	N/A	N/A	N/A
N/A	N/A	N/A	N/A
0.79	0.79	58.3	41.6
N/A	N/A	N/A	N/A
N/A	N/A	N/A	N/A
N/A	N/A	N/A	N/A
0.16	0.06	43.5	10.7
0.11	0.11	25.74	11.33
1.5	N/A	107.3	N/A
N/A	N/A	N/A	N/A
1.6	0.6	94.4	50.13
0.8	0.15	60.15	15.7
N/A	N/A	N/A	N/A
N/A	N/A	N/A	N/A
N/A	N/A	N/A	N/A
1.7	0.59	147.7	53.7
N/A	N/A	N/A	N/A
0.92	0.5	26.29	26.29

*Note*. Rx= Treatment; TB = Total Body.

**Table 7:** Detailed radiological characteristics of the population before and after treatment

DXA			
DXA 1^st^ TB	DXA 1^st^ Spine	DXA post TB	DXA post Spine
N/A	3.2	3.2	4.5
-2.6	N/A	N/A	N/A
N/A	N/A	N/A	N/A
N/A	N/A	N/A	N/A
-1.9	-2.8	N/A	N/A
-2.5	-2.5	N/A	N/A
N/A	N/A	N/A	N/A
N/A	N/A	N/A	N/A
N/A	N/A	N/A	N/A
-1.1	2.1	-0.7	3.2
N/A	N/A	N/A	N/A
N/A	N/A	N/A	N/A
N/A	N/A	N/A	N/A
-1	N/A	N/A	N/A
N/A	N/A	N/A	N/A
N/A	N/A	N/A	N/A
N/A	N/A	N/A	N/A
N/A	N/A	N/A	N/A
N/A	N/A	N/A	N/A
-1.2	-0.1	N/A	N/A
-2.2	-3	N/A	N/A
-0.7	-1.9	N/A	N/A
0.67	N/A	N/A	N/A
-2.2	-0.8	0.3	N/A
N/A	N/A	N/A	N/A
-2.7	-2	-2.6	-1.9
6	N/A	2.5	3.9
N/A	N/A	N/A	N/A
-3.1	-3	N/A	N/A
N/A	-2.4	N/A	-1.6
N/A	N/A	N/A	N/A
N/A	N/A	N/A	N/A
N/A	N/A	N/A	N/A
N/A	-5.5	-6.5	-6.1
N/A	-4.1	-6.2	-4
-2.6	-3	-2.2	-2.5
-6.5	-5.3	N/A	N/A
N/A	-1.3	2.3	1.7
-3	-5.5	N/A	-2
N/A	N/A	N/A	N/A
-3.3	-4.3	N/A	N/A
-4	-4.9	-5	-5
-1.8	-1.8	1.8	1.3
N/A	N/A	N/A	N/A
-2.3	-1.8	N/A	N/A

*Note*. Rx= Treatment; TB = Total Body.

### 2.4 Statistical Analysis

Statistical Package for Social Science (SPSS) V20 was used to measure, examine and analyze the data. Values were expressed as numbers (percentage %) or mean ±Standard deviations. A paired t-test analysis was done, for the assessment of clinical and biochemical efficacy of the drug in the studied group with a 95% confidence interval. Mobility and pain frequency was graded and ranked to determine the mean difference of the change before and after the treatment.

### 2.5 Bibliography

References were managed and cited using EndNotes X7, with American Psychological Association 6^th^ edition (APA 6^th^) style.

## 3. Results

### 3.1 Clinical Characteristic

A total of 46 patients with secondary osteoporosis following in King Abdulaziz University Hospital were collected for the purpose of this study. Thirty-eight of patients have been interviewed retrospectively, while the remaining 8 couldn’t be reached and their data was obtained from the electronic medical record (EMR) with some deficiencies in quality of life, pain and mobility. Out of the total, 18 (39.13%) members of the study group were males and 28 (60.87%) were females. The mean age calculated for the population was 14.76 ± 4.64 years. Twenty patients (43.48%) of the selected group were Saudi and twenty-six (57.52%) were non-Saudi. Consanguinity was reported in eighteen (39.13%) of interviewed patients, twelve (66.67%) were first degree relatives and six (33.33%) were second degree. Seven (21.21%) patients had family history of osteoporosis below the age of 50 years.

The median age of diagnosis was 11 years and the median age of treatment was 14.21 years. Ten (26.32%) of the study subjects had bone deformities. The median number of doses for all the patients was 5 doses. The median weight of population was 34.10 Kg, with a median weight standard deviation (SD) of -0.91 Kg was observed. While, the median height was 140 cm, with median height SD of -2.031 cm. detailed information of the clinical characteristics of the population is shown in Table 1.

### 3.2 Clinical Assessment

Ten (26.32%) of patients in the selected study presented with fractures. There was a statistically significant improvement in the number of fractures after the administration of the zoledronic acid with t (37) =2.96, p=0.005. The mean number of fractures before the treatment was 0.34 ±0.73, which got better after treatment 0.01 ±0.04. Only 2 of the patient who had been treated with zoledronic acid had fractures during their treatment period. Quality of life, mobility, and pain frequency results were related to primary diagnosis.

### 3.3 Quality of Life

Seventeen (44.74%) of the interviewed patients, when asked about their quality of life compared to their peers, subjectively reported a quality of life below normal. After being started on zoledronic acid infusion, eleven (28.95%) reported improvement in quality of life, twenty-six (68.42%) had no change in their quality of life and one (2.63%) reported worse quality of life after treatment.

### 3.4 Pain Frequency

A total of 28 (73.68%) patients presented with bone pain. Both location of pain and frequency were obtained and were provided in Table 4. Using t-test we found out that there was a statistical significance of improvement in pain frequency before and after treatment with t(18)= 4.53 p=0.000 with 95% confidence interval.

### 3.5 Mobility

**Figure 1:**
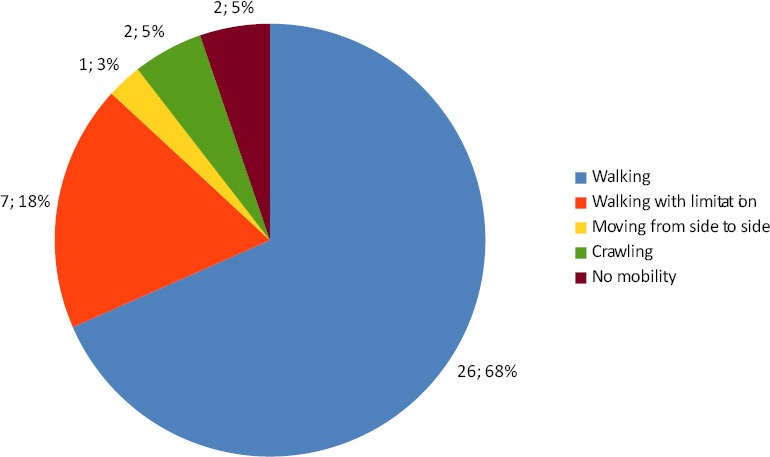
Pie charts representing descriptive statistic for types of mobility before treatment

**Figure 2:**
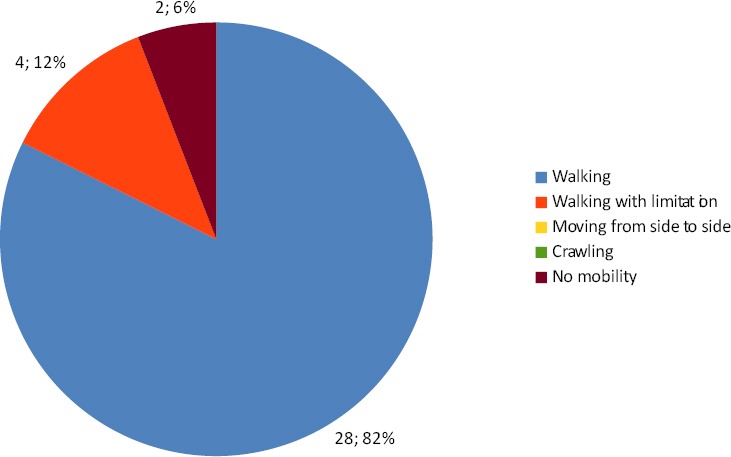
Pie charts representing descriptive statistic for types of mobility after treatment

Regarding the effect of treatment on mobility there was no significant improvement in mobility after therapy, t(33)= 0.70, p=0.488, a descriptive statistic for types of mobility are demonstrated in Figures 1&2.

### 3.6 Clinical Safety

The researchers interviewed 22 who underwent therapy to assess the clinical safety of the drug. After the assessment, 11 (50%) had fever, hypocalcemia occurred in 3 (13.64%), 6 (27.27%) had decreased oral intake, twelve (54.55%) had bone pain and six (28.57%) had flu-like symptoms. Most of these occurred in the first dose.

### 3.7 Biochemical

Mean calcium level before the treatment was 2.22 ± 0.17, the mean of lowest level of calcium was 2.01 ± 0.25. The mean lowest level of phosphate was 0.98 ± 0.41, alkaline phosphatase (ALP) was 132.69 ± 98.7, parathyroid hormone (PTH) was 3.23 ± 2 and the mean of highest level of vitamin D was 77.33 ± 23.75.

The mean value of osteocalcin before and after treatment was 72.52 ±46.96 and 39.53 ±23.33 respectively. While the mean value of CTX before treatment was 1.08 ±0.72 and after treatment was 0.44 ±0.24. There were a significant lowering of both osteocalcin and CTX before and after treatment in 14 cases with p= 0.003 and p= 0.008. Detailed values of osteocalcin and CTX of patients are in Table 5.

## 4. Discussion

Up to the date of publishing this paper, this is the first study following up Zoledronic acid therapy data in children with secondary osteoporosis in the Middle East. This study aims to share our experience with Zoledronic Acid Therapy in Pediatric patients with secondary osteoporosis (in Jeddah, Saudi Arabia). Forty-nine children with secondary osteoporosis were collected, out of those 46 participants received Zoledronic acid therapy in the span of 6 months and up to nine years. The remaining three, their clinical and radiological status were not indicative to initiate therapy. Two-thirds of patients were females and the mean age of presentation is 10 year. Patients’ height and weight were age-matched standard deviation after treatment.

Zoledronic acid is highly effective pharmacological treatment of osteoporosis and considered to be most potent nitrogen-containing bisphosphonates (which also include alendronate, ibandronate, risedronate) and have strongest bone binding affinity which make the cyclic Zoledronic acid infusion (0.25 mg/kg) given in low frequencies (every 3-6 month) intervals in short infusion time (15-60 min) in comparing to other type of bisphosphonates (e.g. pamidronate dose 1mg/kg. d given in three consecutive days every 4 months and take long infusion time (90-120 min)) it’s easier to perform for children and their family (Bachrach & Ward, 2009; Diab & Watts, 2013).

Bone pain was the most common presenting symptom in this study 67.5%. Other symptoms include bone deformity, fracture, and immobility with rates of 25%, 18.37% and 4.08%, respectively. All of these symptoms improved dramatically after the first dose of treatment.

From the first dose of therapy, quality of life and the outcome of management with Zoledronic acid in our patients is highly satisfying with significant decrease in occurrence of new fracture and decrease in bone turnover marker, this result is similar to study published by (Simm et al., 2011), which showed a decrease in fracture rate with no report of fracture event at the time of study and reduced bone turnover markers to the baseline with using the same protocol. In other bisphosphonates like alendronate, the effect of the therapy on fracture rate takes time to appear and reach the point when there is no further significant decrease in fracture rate and bone turnover marker take 3 years to be significantly decreased (Vyskocil, Pikner, & Kutilek, 2005). The improvement in fracture rate was accompanied by reduction in bone pain and improvement in patients’ mobility which is the same finding in all bisphosphonate therapy.

(Watts & Diab, 2010) also carried out a study regarding the long-term use of bisphosphonate therapy in osteoporosis. They illustrated that orally administered bisphosphonates can lead to esophageal irritation, and should not be given to the patients with gastrointestinal problems. Fever, lymphopenia and myalgia are common symptoms observed with bisphosphonate administration. Moreover, iritis and hypocalcemia have been found to be prevalent in patients who were given bisphosphonate to treat osteoporosis. In addition, renal function impairment and renal toxicity might occur in intravenously given bisphosphonates. Bone pain (54.55%) and fever (50%) were the most common acute side effect reported in our patients, only after the first dose of Zoledronic acid infusion. No long-term side effects were reported.

There was a limitation in Bone Mass Density (BMD) assessments as hospital’s DXA scan machine software didn’t support measuring bone density for patients below the age of 5 years as well the frequent machine dysfunction. Also, some baseline DXA went missing as it was done outside the hospital for many of the patients. It was decided not to include any radiological data in this study and we provide some of them in (Table 5). In addition to that we find some difficulty to reach some patient so we decided to obtain their data from the electronic medical record (EMR) with some deficiencies in quality of life, pain and mobility.

## 5. Conclusion

Results of this study support that short and long-term Zoledronic acid therapy is safe and tolerated (with sufficient monitoring for first dose complications). Zoledronic acid therapy was also associated with significant decreased in pain frequency, number of fractures and bone turnover markers (osteocalcin). Acute side-effects recognized in some cases including first dose effects of hypocalcemia, acute ‘flu-like’ reaction, low-grade fever, decrease oral intake and bone pain. A further examination of the clinical efficacy of Zoledronic acid comparing it to pamidronate and alendronate is recommended. We advocate pediatricians’ awareness about preventive measures such as increased physical activity, maintaining mobilization and weight bearing activities and ensuring adequate vitamin D and calcium intake and improve general nutrition.
